# Supplementary material for: Enhancing the diagnosis of functionally relevant coronary artery disease with machine learning
Source: Nat Commun. 2024 Jun 12;15:5034. doi: 10.1038/s41467-024-49390-y (PMC11169272; doi:10.1038/s41467-024-49390-y)
Supplement: Supplementary file 1 — Supplementary Information [file 41467_2024_49390_MOESM1_ESM.pdf]

Supplementary Information for Enhancing the diagnosis of functionally relevant coronary artery disease with machine learning

Christian Bock[1,2,†], Joan Elias Walter[3,4,5,†], Bastian Rieck[1,2,6,†], Ivo Strebel[3,4], Klara Rumora[3,4], Ibrahim Schaefer[3,4], Michael J. Zellweger[3,4], Karsten Borgwardt[1,2,7,‡], Christian Müller[3,4,‡]

[1]Department of Biosystems Science and Engineering, ETH Zürich, Basel, Switzerland. [2]Swiss Institute for Bioinformatics, Lausanne, Switzerland. [3]Cardiovascular Research Institute Basel, University Hospital of Basel, University of Basel, Switzerland. [4]Department of Cardiology, University Hospital of Basel, University of Basel, Switzerland. [5]Department of Endocrinology, Diabetology and Clinical Nutrition, University Hospital Zurich, University of Zurich, Zurich, Switzerland. [6]Institute of AI for Health, Helmholtz Munich and Technical University of Munich, Germany. [7]Department of Machine Learning and Systems Biology, Max Planck Institute of Biochemistry, Martinsried, Germany.

Supplementary Table 1 | Parameter grid for conventional machine learning models. Full maximum depth means nodes are expanded until all leaves are pure. Source data are provided as a Source Data file. To evaluate the classification performance of conventional, linear, and non-linear machine learning models trained on static clinical features alone, we performed grid searches for a logistic regression model, a decision tree classifier, a support vector machine, and a random forest classifier. The best model for each grid search was chosen based on the area under the precision-recall curve. The AUPRC of the results of the grid searches are shown in Supplementary Table 2. We selected the random forest for further investigation due to its superior performance on the validation cohort.

Model	Parameter	Values
Support Vector Machine	Kernel	{linear, polynomial, RBF, sigmoid}
	C	{1E-2, 0.1, 1, 10}
	Degree for polynomial kernel	{1,2, 3}
	Gamma	{1E-6, 3.2E-2, ⅛, 1000}
	Coefficient for polynomial and sigmoid kernel	{0, 0.4, 0.7, 1}
	Shrinking	{true, false}
	Tolerance for stopping criterion	{1E-4, 1E-3, 1E-2}
Logistic Regression	Penalty	{none, l1, l2, elastic net}
	C	{1E-2, 0.1, 1, 5, 10}
	Tolerance	{1E-4, 1E-3, 1E-2}
	Class weight	{balanced, none}
	Fit intercept	{true, false}
Decision Tree	Splitter	{best, random}
	Criterion	{gini, entropy}
	Maximum depth	{2, 5, 10, 20, 50, full}
	Minimum samples split	{1, 2, 5, 10, 15}
Random Forest	Number trees	{25, 50, 100}
	Maximum number of features	{2, 5, 8}
	Maximum depth	{full, 5, 10}
	Minimum impurity increase	{1E-5, 1E-4, 1E-3}

Supplementary Table 2 | Results of the grid searches for the statistical machine learning approaches. Parameters were selected based on the best validation performance. Source data are provided as a Source Data file.

Model	Validation AUPRC
Support Vector Machine	0.38 ± 0.05
Logistic Regression	0.49 ± 0.03
Decision Tree	0.57 ± 0.08
**Random Forest**	**0.61 ± 0.04**

Supplementary Table 3 | Architectural details. Convolutional layers are written as [input dimension, output dimension, kernel size, stride]Conv, linear layers as [input dimension, output dimension]Lin. BN: Batch norm, ReLU: Rectified Linear Unit, DO: Dropout. Max pooling is written as MP(kernel size; stride). add denotes the addition of the output of the MP_{1x1} layer and the preceding convolutional layer. Source data are provided as a Source Data file.

Task	Layer Name	Parameters
N/A	MP_{1x1}	[MP(4, 4), $[64, 128, 1, 1]_{Conv}$]
N/A	BR	[BN, ReLU]
N/A	BRD	[BR, DO(0.8)]
N/A	$Conv_{init}$	[$[1, 64, 20, 1]_{Conv}$, BR]
N/A	Res_1	[$[64, 128, 20, 1]_{Conv}$, BRD, $[128, 128, 20, 4]_{Conv}$, add, BRD]
N/A	Res_2	[$[128, 196, 20, 1]_{Conv}$, BRD, $[196, 196, 20, 4]_{Conv}$, add, BRD]
N/A	Res_3	[$[196, 256, 20, 1]_{Conv}$, BRD, $[256, 256, 20, 4]_{Conv}$, add, BRD]
	Res_4	[$[320, 320, 20, 1]_{Conv}$, BRD, $[320, 320, 20, 5]_{Conv}$, add, BRD]
N/A	Res_5	[$[320, 160, 20, 1]_{Conv}$, BRD, $[160, 160, 20, 4]_{Conv}$, add, BRD]
Embedding ECG	h_{res}	[$Conv_{init}$, Res1, Res2, Res3, Res4, Res5, BR]
fCAD Prediction	g_{lin}	[$[672, 32]_{Lin}$, ReLU, DO(0.5), $[32, 1]_{Lin}$]
MPSSRS Prediction	g_{lin}	[$[672, 32]_{Lin}$; ReLU, DO(0.4), $[32, 1]_{Lin}$]
MPSSSS Prediction	g_{lin}	[$[672, 32]_{Lin}$; ReLU, DO(0.4), [32, 1]Lin]
Stress Type Prediction	g_{lin}	[$[672, 32]_{Lin}$; ReLU, DO(0.4), $[32, 5]_{Lin}$]
Embedding Clinical Features	h_{lin}	[$[8, 16]_{Lin}$, ReLU, BN, $[16, 32]_{Lin}$, ReLU, BN, DO(0.5)]

Supplementary Table 4 | Multi-task regularisation grid. Parameter grid to determine multi-task regularisation parameters. η_{best} refers to the best learning rate from the first selection step. Source data are provided as a Source Data file.

Parameter	Values
λ_{MPSSRS}	{0.00, 0.25, 0.50, 0.75, 1.00}
λ_{MPSSSS}	{0.00, 0.25, 0.50, 0.75, 1.00}
λ_{Stress}	{0.00, 0.25, 0.50, 0.75, 1.00}
η	$\{2\eta_{best}, \eta_{best}, \frac{\eta_{best}}{2}\}$

Supplementary Table 5 | Multi-task regularisation results. Impact of regularisation strength on mean AUPRC (%) on the validation sets over all splits and learning rates. Uncertainty is shown as standard deviation. None refers to training without any regularisation, Best to the configuration with highest mean AUPRC over all five validation splits. Highest mean AUPRC over all validation sets is reached on lead V6 with $\lambda_{MPSSRS} = \lambda_{MPSSSS} = 0.5$, and $\lambda_{Stress} = 0.75$. Source data are provided as a Source Data file.

Regularisation Strength		Lead aVR	Lead V1	Lead V6
λ_{MPSSRS}	0.00	54.71 ± 1.73	52.47 ± 1.01	55.94 ± 1.26
	0.25	55.55 ±0.87	52.99 ± 0.45	56.57 ± 0.60
	0.50	55.59 ±0.86	52.93 ± 0.46	56.70 ± 0.50
	0.75	55.56 ±0.86	52.93 ± 0.48	56.81 ± 0.45
	1.00	55.26 ±1.07	52.85 ± 0.48	56.80 ± 0.47
λ_{MPSSSS}	0.00	53.91 ±1.36	52.07 ± 1.01	55.86 ± 1.18
	0.25	55.56 ±0.88	53.03 ± 0.40	56.82 ± 0.53
	0.50	55.82 ±0.75	53.10 ± 0.39	56.90 ± 0.48
	0.75	55.71 ±0.77	53.04 ± 0.38	56.68 ± 0.48
	1.00	55.66 ±0.77	52.93 ± 0.40	56.54 ± 0.54
λ_{Stress}	0.00	54.82 ±1.05	52.64 ±0.64	56.10 ± 0.76
	0.25	55.37 ±1.12	52.77 ±0.61	56.45 ± 0.73
	0.50	55.36 ±1.16	52.88 ± 0.63	56.70 ± 0.62
	0.75	55.54 ±1.17	52.89 ±0.62	56.74 ± 0.85
	1.00	55.57 ±1.23	52.98 ±0.67	56.82 ± 0.75
None		51.21 ±0.17	50.73 ±0.58	53.80 ± 0.21
Best				57.23 ± 0.68

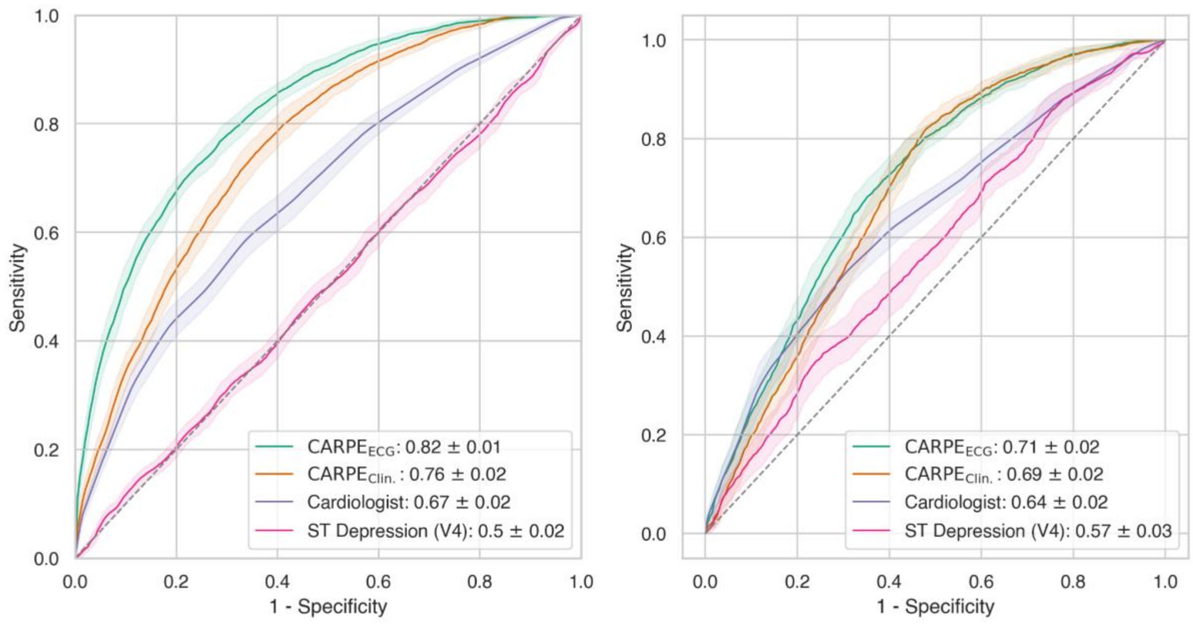

Supplementary Figure 1 | **Overfitting Assessment:** Receiver operating characteristics curves of the treating physician's clinical assessment prediction (Cardiologist), $\text{CARPE}_{\text{ECG}}$, $\text{CARPE}_{\text{Clin.}}$, and ST depression in the training (right) and held-out test cohorts (left). Numbers in legend are area under the curve and their standard deviations. While there is a performance drop from training to test data in all approaches except for the ST depression approach, it is most prominent in $\text{CARPE}_{\text{ECG}}$.

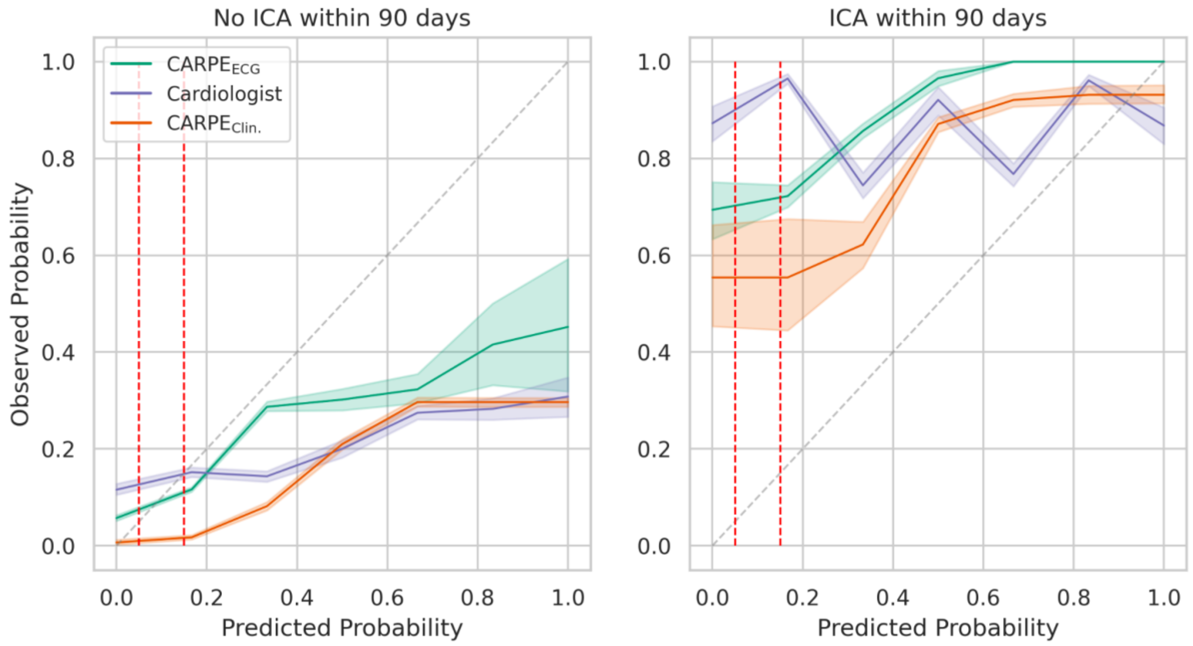

Supplementary Figure 2 | Calibration plot of patients with and without ICA within 90 days of ECG stress test. Red dotted line marking decision thresholds at 5% and 15%. Source data are provided as a Source Data file.

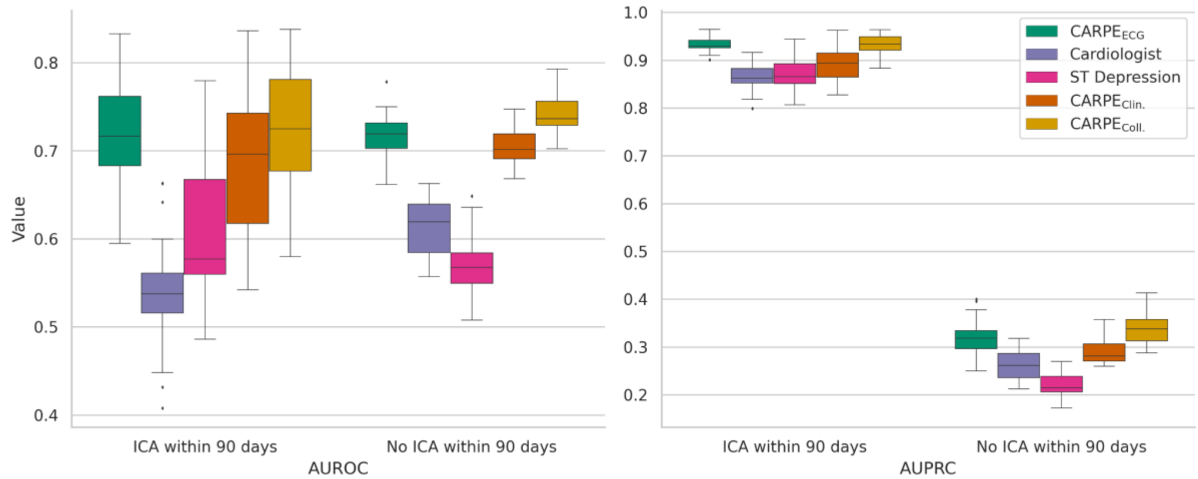

Supplementary Figure 3 | Predictive performance (AUROC and AUPRC) for patients with and without ICA. Source data are provided as a Source Data file.

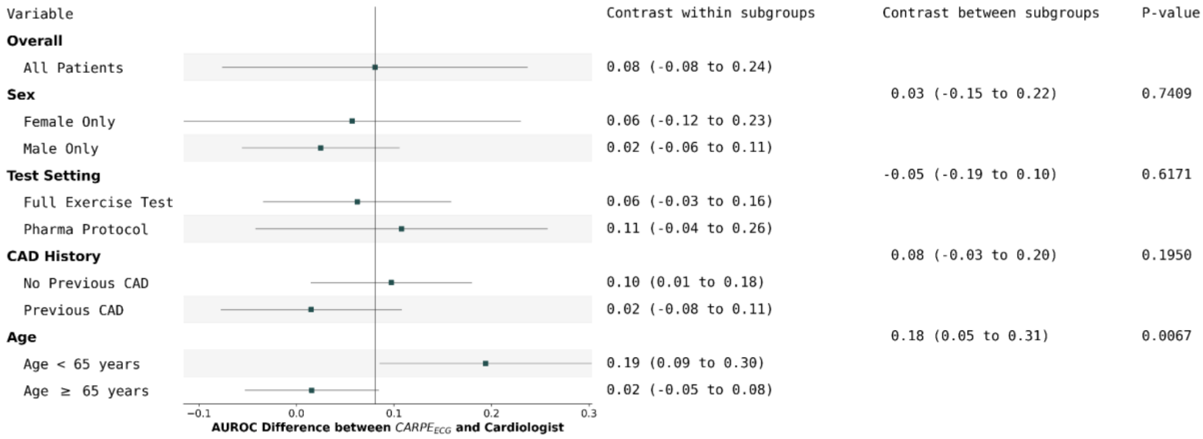

Supplementary Figure 4 | Interaction analysis showing the statistical interaction of four subgroups. The "Age" variable is the only feature exhibiting a statistical effect. Rectangles show means; bars show the 95% confidence intervals. Source data are provided as a Source Data file. Source data are provided as a Source Data file.

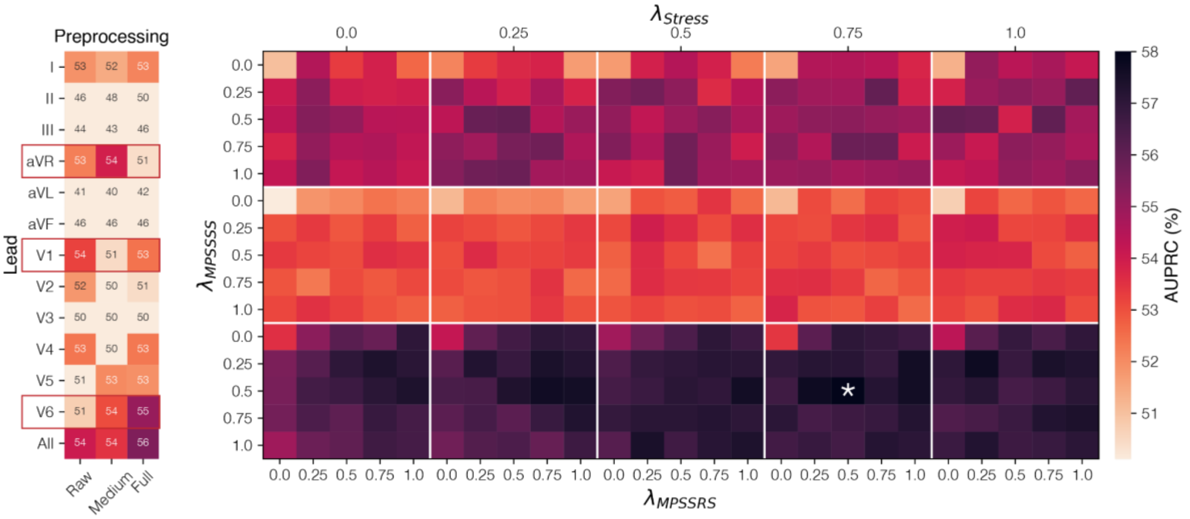

Supplementary Figure 5 | **Preprocessing and lead selection.** Performance heatmaps for lead, preprocessing, and regularisation parameter selection. Prevalence: 34%. Left: Best AUPRC among three learning rates per lead and preprocessing pipeline. The best three leads are highlighted with a red rectangle. Right: Results of the grid search to find the best regularisation parameters. Large rows (separated by white horizontal lines) represent the three best-performing leads (aVR, V1, and V6, respectively). Large columns (separated by white vertical lines) represent five settings for λ_{Stress} (upper x-axis), small columns and rows respective regularisation values for λ_{MPSSRS} and λ_{MPSSSS}. The best regularisation combination is marked with a white asterisk. Source data are provided as a Source Data file.

Supplementary Table 6 | Diagnostic Performance Subcohort Analysis. Performance analysis on different cohorts, i.e. (1) all patients, (2) patients that completed the stress test on the bicycle, (3) patients on a pharmacological protocol, (4) patients without CAD history, (5) patients with a history of CAD, (6/7) female/male patients, (8) patients younger than 65 years, and (9) patients that are 65 years or older. Prevalence values are provided as they represent the AUPRC a random classifier would reach. Asterisks indicate a statistically significant effect of the respective computational approach compared to the performance of the cardiologist. Diamonds indicate a statistically significant effect compared to $CARPE_{ECG}$.p-values are computed using a one-sided Kolmogorov-Smirnov test. Multiple hypotheses are corrected for using Bonferroni correction. Number of stars/diamonds signal strength of statistical effect: One symbol: p<0.05/correction factor, two symbols: p<0.01/correction factor, three symbols: p<0.001/correction factor. The correction factor equals the number of subgroups (ten) multiplied by the number of comparisons (three for the comparison with the cardiologist, one for the comparison with $CARPE_{ECG}$). Source data are provided as a Source Data file.

Cohort	Method	AUROC ± STD	AUPRC ± STD
All Patients, (n=803)<br>Prevalence: 28.3%	$CARPE_{ECG}$	0.71 ± 0.02*** , p=4.0e-13	0.46 ± 0.03* , p=9.6e-04
	Cardiologist	0.64 ± 0.02	0.42 ± 0.03
	ST Depression (lead V4)	0.58 ± 0.02, p=1.0	0.34 ± 0.02, p=1.0
	$CARPE_{Clin.}$	0.70 ± 0.02***, p=1.6e-10	0.42 ± 0.02, p=5.33e-01
	$CARPE_{Coll.}$	**0.74 ± 0.03$^{\diamond\diamond\diamond}$, p=7.9e-07**	**0.49 ± 0.03$^{\diamond\diamond\diamond}$, p=7.9e-07**
Full Exercise Testing, (n=482)<br>Prevalence: 24.5%	$CARPE_{ECG}$	0.74 ± 0.03*** , p=7.9e-07	0.47 ± 0.03, p=0.00281
	Cardiologist	0.68 ± 0.03	0.42 ± 0.04
	ST Depression (lead V4)	0.58 ± 0.04, p=1.0	0.31 ± 0.03, p=1.0
	$CARPE_{Clin.}$	0.73 ± 0.03** , p=8.1e-05	0.41 ± 0.04, p=0.7021
	$CARPE_{Coll.}$	**0.77 ± 0.03,** p=7.42e-03	**0.52 ± 0.04**$^{\diamond\diamond\diamond}$, p=4.2e-06
Pharmacological Testing, (n=100)<br>Prevalence: 33.0%	$CARPE_{ECG}$	0.69 ± 0.07** , p=8.1e-05	0.47 ± 0.01, p=0.0178
	Cardiologist	0.58 ± 0.07	0.40 ± 0.06
	ST Depression (lead II)	0.56 ± 0.07, p=0.9615	0.39 ± 0.06, p=0.8547
	$CARPE_{Clin.}$	0.65 ± 0.07, p=0.0178	0.42 ± 0.06, p=0.1428
	$CARPE_{Coll.}$	**0.70 ± 0.07,** p=0.3728	**0.50 ± 0.10,** p=0.1428
No prior history of CAD, (n=446)<br>Prevalence: 20.6%	$CARPE_{ECG}$	0.73 ± 0.03***, p=9.7e-12	0.38 ± 0.04***, p=4.2e-06
	Cardiologist	0.63 ± 0.04	0.33 ± 0.03
	ST Depression (lead V4)	0.52 ± 0.03, p=1.0	0.24 ± 0.03, p=1.0
	$CARPE_{Clin.}$	0.73 ± 0.04***, p=9.7e-12	0.37 ± 0.05, p=0.0028
	$CARPE_{Coll.}$	**0.75 ± 0.04**, p=7.42e-03	**0.44 ± 0.05**$^{\diamond\diamond}$, p=8.1e-05
	CADConsortium	0.65 ± 0.03, p=7.78e-02	0.32 ± 0.04, p=0.8547
	ESC2019	0.68 ± 0.04**, p=2.95e-04	0.32 ± 0.03, p=0.8547
Prior history of CAD, (n=357)<br>Prevalence: 37.8%	$CARPE_{ECG}$	0.64 ± 0.03, p=0.2405	0.51 ± 0.04, p=0.3728
	Cardiologist	0.63 ± 0.04	0.49 ± 0.04
	ST Depression (lead V4)	0.63 ± 0.04, p=0.7021	0.48 ± 0.04, p=0.8547
	$CARPE_{Clin.}$	0.58 ± 0.03, p=1.0	0.44 ± 0.03, p=1.0
	$CARPE_{Coll.}$	**0.68 ± 0.03,** p=0.0178	**0.53 ± 0.04,** p=0.0779
Female Only, (n=272)<br>Prevalence: 12.9%	$CARPE_{ECG}$	**0.69 ± 0.06,** p=0.0028	0.26 ± 0.06, p=0.3728
	Cardiologist	0.63 ± 0.07	0.24 ± 0.06
	ST Depression (lead V4)	0.50 ± 0.04, p=1.0	0.13 ± 0.02, p=1.0
	$CARPE_{Clin.}$	0.68 ± 0.07, p=0.0074	0.25 ± 0.07, p=0.5326
	$CARPE_{Coll.}$	**0.69 ± 0.06,** p=0.5326	**0.29 ± 0.06,** p=0.2405
Male Only, (n=531)<br>Prevalence: 36.2%	$CARPE_{ECG}$	0.67 ± 0.03, p=0.0074	0.50 ± 0.04, p=0.1428
	Cardiologist	0.64 ± 0.03	0.49 ± 0.04
	ST Depression	0.57 ± 0.04, p=1.0	0.41 ± 0.03, p=1.0
	$CARPE_{Clin.}$	0.60 ± 0.04, p=1.0	0.43 ± 0.03, p=1.0
	$CARPE_{Coll.}$	**0.69 ± 0.03,** p=0.0178	**0.53 ± 0.04,** p=0.0390
Age < 65 years, (n=292)<br>Prevalence: 20.5%	$CARPE_{ECG}$	0.78 ± 0.04***, p=7.9e-15	0.45 ± 0.06***, p=9.7e-12
	Cardiologist	0.59 ± 0.05	0.30 ± 0.04
	ST Depression	0.59 ± 0.05, p=0.7021	0.28 ± 0.05, p=0.8547
	$CARPE_{Clin.}$	0.74 ± 0.04***, p=4.0e-13	0.39 ± 0.05***, p=1.3e-07
	$CARPE_{Coll.}$	**0.79 ± 0.04,** p=0.2405	**0.47 ± 0.05,** p=0.3728
Age ≥ 65 years, (n=511)<br>Prevalence: 32.7%	$CARPE_{ECG}$	0.66 ± 0.03, p=0.1428	0.46 ± 0.04, p=0.8547
	Cardiologist	0.65 ± 0.02	0.47 ± 0.03
	ST Depression	0.58 ± 0.02, p=1.0	0.40 ± 0.03, p=1.0
	$CARPE_{Clin.}$	0.64 ± 0.03, p=0.8547	0.41 ± 0.03, p=1.0
	$CARPE_{Coll.}$	**0.69 ± 0.02**$^{\diamond\diamond\diamond}$, p=8.1e-05	**0.49 ± 0.04,** p=0.0178
Age < 65 years and full exercise test, (n=214)<br>Prevalence: 16.8%	$CARPE_{ECG}$	0.78 ± 0.04***, p=9.7e-12	0.40 ± 0.06***, p=7.9e-07
	Cardiologist	0.62 ± 0.06	0.27 ± 0.06
	ST Depression	0.66 ± 0.06, p=7.79e-02	0.28 ± 0.06, p=0.0241
	**$CARPE_{Clin.}$**	0.76 ± 0.04***, p=1.6e-10	0.33 ± 0.05**, p=3.0e-04
	$CARPE_{Coll.}$	**0.79 ± 0.04**, p=0.1428	**0.43 ± 0.07**, p=0.2405

Supplementary Table 7 | Diagnostic Performance on External Validation. Area under receiver operator characteristic and precision recall curve on the internal (held-out) data set and the external validation set. Values in parentheses are computed on an upsampled data set to match the prevalence of the internal validation of 24.5% such that AUPRC values are comparable. Source data are provided as a Source Data file.

	Internal Validation (exercise stress only) fCAD prevalence: 24.5%		External Validation fCAD prevalence: 7.5%	
	AUPRC ± STD	AUROC ± STD	AUPRC ± STD	AUROC ± STD
$CARPE_{Clin.}$	0.41 ± 0.04	0.73 ± 0.03	0.19 ± 0.01 (0.47 ± 0.03)	0.75 ± 0.004 (0.75 ± 0.02)
**$CARPE_{ECG}$**	**0.47 ± 0.03**	**0.74 ± 0.03**	**0.28 ± 0.02 (0.58 ± 0.03)**	**0.80 ± 0.01 (0.80 ± 0.01)**

Supplementary Table 8 | Additional Performance Metrics. Positive predictive value (PPV), specificity, F1 score, and accuracy score at the three decision thresholds recommended in international guidelines for different subcohorts. The score distribution may lead to results with zero standard deviation. Highest mean values are highlighted in bold. Source data are provided as a Source Data file.

**Threshold <5% for rule-out**					
	Method	PPV ± STD	Specificity ± STD	F1 Score ± STD	Accuracy ± STD
All patients (n=803) Prevalence: 28.3%	$CARPE_{Coll.}$	0.29 ± 0.003	0.07 ± 0.012	0.45 ± 0.004	0.33 ± 0.009
	$CARPE_{ECG}$	**0.31 ± 0.005**	**0.14 ± 0.020**	**0.47 ± 0.006**	**0.38 ± 0.014**
	$CARPE_{Clin.}$	0.28 ± 0.000	0.00 ± 0.000	0.44 ± 0.000	0.28 ± 0.000
	Cardiologist	0.29 ± 0.003	0.05 ± 0.011	0.45 ± 0.004	0.32 ± 0.008
No prior CAD (n=446) Prevalence: 20.6%	$CARPE_{Coll.}$	0.22 ± 0.005	0.11 ± 0.017	0.36 ± 0.007	0.29 ± 0.015
	$CARPE_{ECG}$	**0.24 ± 0.007**	**0.21 ± 0.025**	**0.38 ± 0.011**	**0.37 ± 0.021**
	$CARPE_{Clin.}$	0.21 ± 0.000	0.00 ± 0.000	0.34 ± 0.000	0.21 ± 0.000
	Cardiologist	0.21 ± 0.003	0.05 ± 0.013	0.34 ± 0.005	0.24 ± 0.010
Prior CAD (n=357) Prevalence: 37.8%	$CARPE_{Coll.}$	0.38 ± 0.000	0.00 ± 0.000	0.55 ± 0.000	0.38 ± 0.000
	$CARPE_{ECG}$	0.38 ± 0.001	0.002 ± 0.003	0.55 ± 0.001	0.38 ± 0.002
	$CARPE_{Clin.}$	0.38 ± 0.000	0.00 ± 0.000	0.55 ± 0.000	0.38 ± 0.000
	Cardiologist	**0.38 ± 0.005**	**0.05 ± 0.018**	**0.55 ± 0.006**	**0.40 ± 0.011**
Female Only, (n=272) Prevalence: 12.9%	$CARPE_{Coll.}$	0.13 ± 0.008	0.14 ± 0.025	0.23 ± 0.013	0.24 ± 0.023
	$CARPE_{ECG}$	**0.15 ± 0.009**	**0.24 ± 0.031**	**0.26 ± 0.015**	**0.33 ± 0.030**
	$CARPE_{Clin.}$	0.12 ± 0.000	0.00 ± 0.000	0.22 ± 0.000	0.12 ± 0.000
	Cardiologist	0.12 ± 0.006	0.03 ± 0.012	0.21 ± 0.010	0.14 ± 0.001
Male Only, (n=531) Prevalence: 36.2%	$CARPE_{Coll.}$	0.36 ± 0.001	0.01 ± 0.006	0.53 ± 0.001	0.37 ± 0.004
	$CARPE_{ECG}$	**0.37 ± 0.004**	0.05 ± 0.011	**0.54 ± 0.005**	0.39 ± 0.008
	$CARPE_{Clin.}$	0.36 ± 0.000	0.00 ± 0.000	0.53 ± 0.000	0.36 ± 0.000
	Cardiologist	**0.37 ± 0.004**	**0.07 ± 0.015**	**0.54 ± 0.005**	**0.40 ± 0.010**
Age < 65 years, (n=292) Prevalence: 20.5%	$CARPE_{Coll.}$	0.22 ± 0.006	0.10 ± 0.029	0.36 ± 0.008	0.28 ± 0.023
	$CARPE_{ECG}$	**0.24 ± 0.009**	**0.21 ± 0.033**	**0.39 ± 0.013**	**0.37 ± 0.028**
	$CARPE_{Clin.}$	0.20 ± 0.001	0.00 ± 0.000	0.34 ± 0.000	0.20 ± 0.002
	Cardiologist	0.21 ± 0.004	0.06 ± 0.012	0.34 ± 0.007	0.25 ± 0.011
Age < 65 years and full exercise test, (n=214) Prevalence: 16.8%	$CARPE_{Coll.}$	0.18 ± 0.005	0.13 ± 0.030	0.31 ± 0.008	0.28 ± 0.025
	$CARPE_{ECG}$	**0.21 ± 0.011**	**0.26 ± 0.040**	**0.34 ± 0.017**	**0.38 ± 0.035**
	$CARPE_{Clin.}$	0.16 ± 0.000	0.00 ± 0.000	0.28 ± 0.000	0.16 ± 0.000
	Cardiologist	0.17 ± 0.006	0.09 ± 0.020	0.30 ± 0.009	0.23 ± 0.018
**Threshold <10% for rule-out**					
All patients (n=803) Prevalence: 28.3%	$CARPE_{Coll.}$	**0.34 ± 0.006**	0.27 ± 0.020	**0.50 ± 0.007**	0.46 ± 0.014
	$CARPE_{ECG}$	**0.34 ± 0.006**	**0.28 ± 0.017**	0.50 ± 0.008	**0.46 ± 0.013**
	$CARPE_{Clin.}$	0.28 ± 0.000	0.00 ± 0.000	0.44 ± 0.000	0.28 ± 0.000
	Cardiologist	0.29 ± 0.003	0.07 ± 0.011	0.45 ± 0.004	0.32 ± 0.008
No prior CAD (n=446) Prevalence: 20.6%	$CARPE_{Coll.}$	**0.29 ± 0.012**	0.42 ± 0.026	**0.43 ± 0.017**	0.52 ± 0.022
	$CARPE_{ECG}$	**0.29 ± 0.015**	**0.44 ± 0.030**	**0.43 ± 0.021**	**0.53 ± 0.026**
	$CARPE_{Clin.}$	0.21 ± 0.000	0.00 ± 0.000	0.34 ± 0.000	0.21 ± 0.000
	Cardiologist	0.21 ± 0.004	0.07 ± 0.015	0.34 ± 0.007	0.25 ± 0.012
Prior CAD (n=357) Prevalence: 37.8%	$CARPE_{Coll.}$	0.38 ± 0.002	0.01 ± 0.009	0.55 ± 0.002	0.38 ± 0.005
	$CARPE_{ECG}$	0.38 ± 0.003	0.02 ± 0.010	0.55 ± 0.004	0.38 ± 0.007
	$CARPE_{Clin.}$	0.38 ± 0.000	0.00 ± 0.000	0.55 ± 0.000	0.38 ± 0.000
	Cardiologist	**0.39 ± 0.005**	**0.07 ± 0.018**	**0.55 ± 0.007**	**0.41 ± 0.012**
Female Only, (n=272)	$CARPE_{Coll.}$	**0.18 ± 0.019**	**0.48 ± 0.034**	**0.29 ± 0.031**	**0.52 ± 0.033**
	$CARPE_{ECG}$	**0.18 ± 0.02**	0.46 ± 0.036	**0.29 ± 0.032**	0.50 ± 0.036
	$CARPE_{Clin.}$	0.12 ± 0.000	0.00 ± 0.000	0.22 ± 0.000	0.12 ± 0.000

Prevalence: 12.9%	Cardiologist	0.12 ± 0.006	0.03 ± 0.012	0.21 ± 0.010	0.14 ± 0.010
Male Only, (n=531) Prevalence: 36.2%	CARPE$_{Coll.}$	0.38 ± 0.006	0.11 ± 0.017	0.55 ± 0.007	0.42 ± 0.012
	CARPE$_{ECG}$	**0.39 ± 0.007**	**0.14 ± 0.018**	**0.56 ± 0.009**	**0.44 ± 0.014**
	CARPE$_{Clin.}$	0.36 ± 0.000	0.00 ± 0.000	0.53 ± 0.000	0.36 ± 0.000
	Cardiologist	0.38 ± 0.005	0.08 ± 0.017	0.54 ± 0.006	0.40 ± 0.012
Age < 65 years, (n=292) Prevalence: 20.5%	CARPE$_{Coll.}$	0.27 ± 0.011	0.36 ± 0.035	0.43 ± 0.014	0.48 ± 0.027
	CARPE$_{ECG}$	**0.28 ± 0.011**	**0.37 ± 0.035**	**0.43 ± 0.014**	**0.49 ± 0.027**
	CARPE$_{Clin.}$	0.20 ± 0.000	0.00 ± 0.000	0.34 ± 0.000	0.20 ± 0.002
	Cardiologist	0.21 ± 0.005	0.08 ± 0.014	0.34 ± 0.008	0.25 ± 0.011
Age < 65 years and full exercise test, (n=214) Prevalence: 16.8%	CARPE$_{Coll.}$	0.24 ± 0.019	0.43 ± 0.048	0.39 ± 0.027	0.51 ± 0.043
	CARPE$_{ECG}$	**0.26 ± 0.020**	**0.47 ± 0.043**	**0.40 ± 0.028**	**0.55 ± 0.038**
	CARPE$_{Clin.}$	0.16 ± 0.000	0.00 ± 0.000	0.28 ± 0.000	0.16 ± 0.00
	Cardiologist	0.18 ± 0.006	0.11 ± 0.025	0.30 ± 0.009	0.25 ± 0.021
**Threshold <15% for rule-out**					
All patients (n=803) Prevalence: 28.3%	CARPE$_{Coll.}$	**0.37 ± 0.007**	0.41 ± 0.021	**0.53 ± 0.009**	**0.55 ± 0.013**
	CARPE$_{ECG}$	0.37 ± 0.008	**0.39 ± 0.020**	0.52 ± 0.009	0.53 ± 0.013
	CARPE$_{Clin.}$	0.28 ± 0.001	0.01 ± 0.004	0.44 ± 0.001	0.29 ± 0.003
	Cardiologist	0.31 ± 0.008	0.25 ± 0.015	0.46 ± 0.012	0.42 ± 0.014
No prior CAD (n=446) Prevalence: 20.6%	CARPE$_{Coll.}$	**0.34 ± 0.024**	**0.63 ± 0.026**	**0.47 ± 0.035**	**0.65 ± 0.023**
	CARPE$_{ECG}$	0.32 ± 0.023	0.59 ± 0.025	0.45 ± 0.033	0.62 ± 0.024
	CARPE$_{Clin.}$	0.21 ± 0.001	0.12 ± 0.007	0.34 ± 0.002	0.22 ± 0.005
	Cardiologist	0.23 ± 0.010	0.27 ± 0.024	0.36 ± 0.015	0.39 ± 0.018
Prior CAD (n=357) Prevalence: 37.8%	CARPE$_{Coll.}$	0.39 ± 0.006	0.06 ± 0.018	**0.56 ± 0.007**	0.41 ± 0.013
	CARPE$_{ECG}$	0.39 ± 0.006	0.15 ± 0.062	**0.56 ± 0.007**	0.41 ± 0.014
	CARPE$_{Clin.}$	0.38 ± 0.000	0.00 ± 0.000	0.55 ± 0.000	0.38 ± 0.000
	Cardiologist	**0.40 ± 0.013**	**0.23 ± 0.030**	0.55 ± 0.017	**0.47 ± 0.023**
Female Only, (n=272) Prevalence: 12.9%	CARPE$_{Coll.}$	**0.21 ± 0.040**	**0.69 ± 0.043**	**0.30 ± 0.056**	**0.67 ± 0.044**
	CARPE$_{ECG}$	0.19 ± 0.029	0.60 ± 0.039	0.29 ± 0.044	0.60 ± 0.040
	CARPE$_{Clin.}$	0.13 ± 0.001	0.02 ± 0.012	0.23 ± 0.002	0.14 ± 0.011
	Cardiologist	0.14 ± 0.014	0.26 ± 0.038	0.23 ± 0.024	0.33 ± 0.035
Male Only, (n=531) Prevalence: 36.2%	CARPE$_{Coll.}$	0.40 ± 0.007	0.21 ± 0.022	**0.57 ± 0.009**	0.48 ± 0.014
	CARPE$_{ECG}$	**0.41 ± 0.009**	**0.24 ± 0.019**	**0.57 ± 0.012**	**0.49 ± 0.016**
	CARPE$_{Clin.}$	0.36 ± 0.000	0.00 ± 0.000	0.53 ± 0.000	0.36 ± 0.000
	Cardiologist	0.39 ± 0.009	0.24 ± 0.020	0.54 ± 0.012	0.47 ± 0.015
Age < 65 years, (n=292) Prevalence: 20.5%	CARPE$_{Coll.}$	0.31 ± 0.022	**0.50 ± 0.038**	0.45 ± 0.029	0.58 ± 0.032
	CARPE$_{ECG}$	**0.31 ± 0.018**	0.49 ± 0.039	**0.47 ± 0.022**	**0.58 ± 0.029**
	CARPE$_{Clin.}$	0.20 ± 0.001	0.02 ± 0.006	0.34 ± 0.001	0.21 ± 0.005
	Cardiologist	0.23 ± 0.018	0.30 ± 0.033	0.36 ± 0.028	0.41 ± 0.033
Age < 65 years and full exercise test, (n=214) Prevalence: 16.8%	CARPE$_{Coll.}$	0.28 ± 0.034	**0.60 ± 0.044**	0.42 ± 0.047	0.63 ± 0.043
	CARPE$_{ECG}$	**0.30 ± 0.029**	0.59 ± 0.045	**0.44 ± 0.038**	**0.64 ± 0.040**
	CARPE$_{Clin.}$	0.17 ± 0.001	0.02 ± 0.020	0.29 ± 0.002	0.18 ± 0.009
	Cardiologist	0.21 ± 0.013	0.35 ± 0.034	0.34 ± 0.021	0.44 ± 0.030

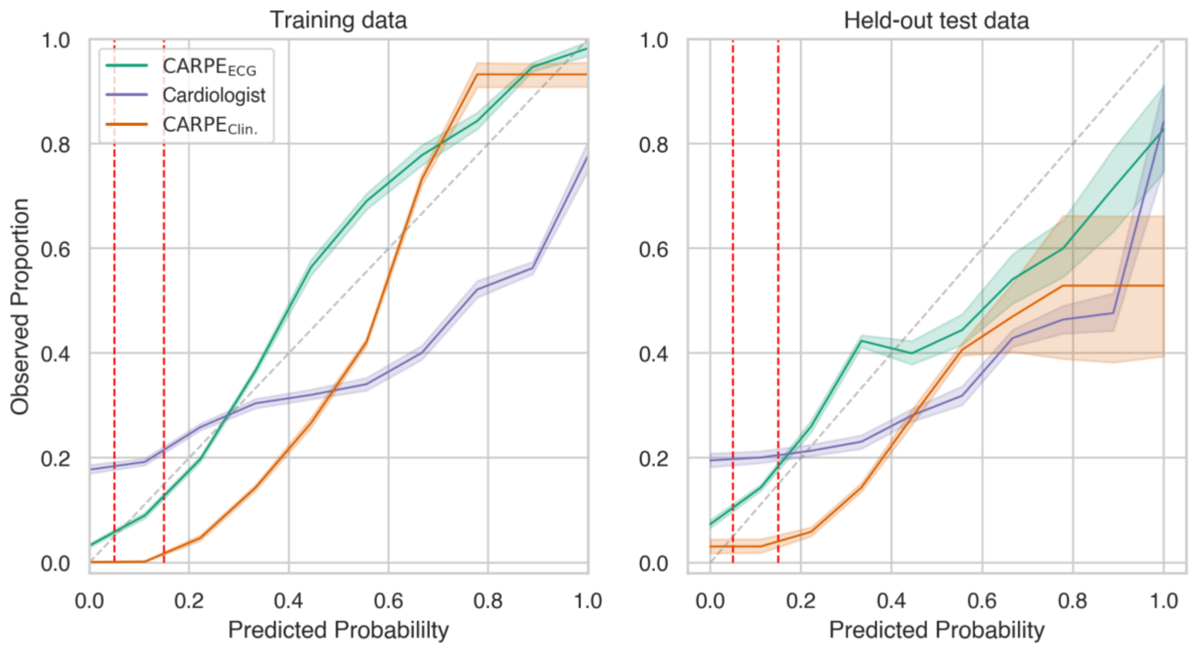

Supplementary Figure 6 | Calibration Plot. Calibration behaviour of CARPE$_{ECG}$, Cardiologist, and CARPE$_{Clin.}$ on both training and held-out test set. A perfectly calibrated classifier is shown in grey. Envelopes show 95% confidence intervals. Advocated decision thresholds are shown as dashed red vertical lines. Source data are provided as a Source Data file.

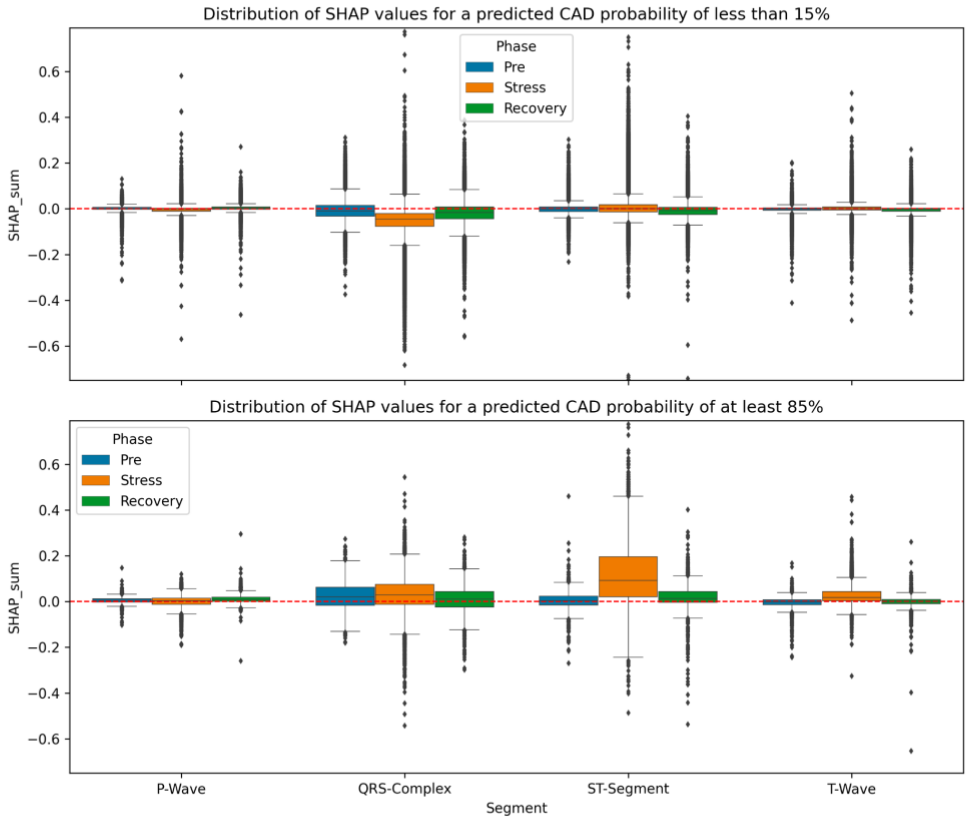

Supplementary Figure 7 | SHAP Analysis of EKG Segments. Aggregated SHAP values stratified by stress phase and segment in populations of low (upper plot) and high (lower plot) predicted CAD risk. Deviations from zero indicate higher contributions to the predicted risk score. On average, ST-segments from the stress phase are most relevant for higher risk scores, and QRS complexes from the stress phase for the prediction of lower scores. Source data are provided as a Source Data file.

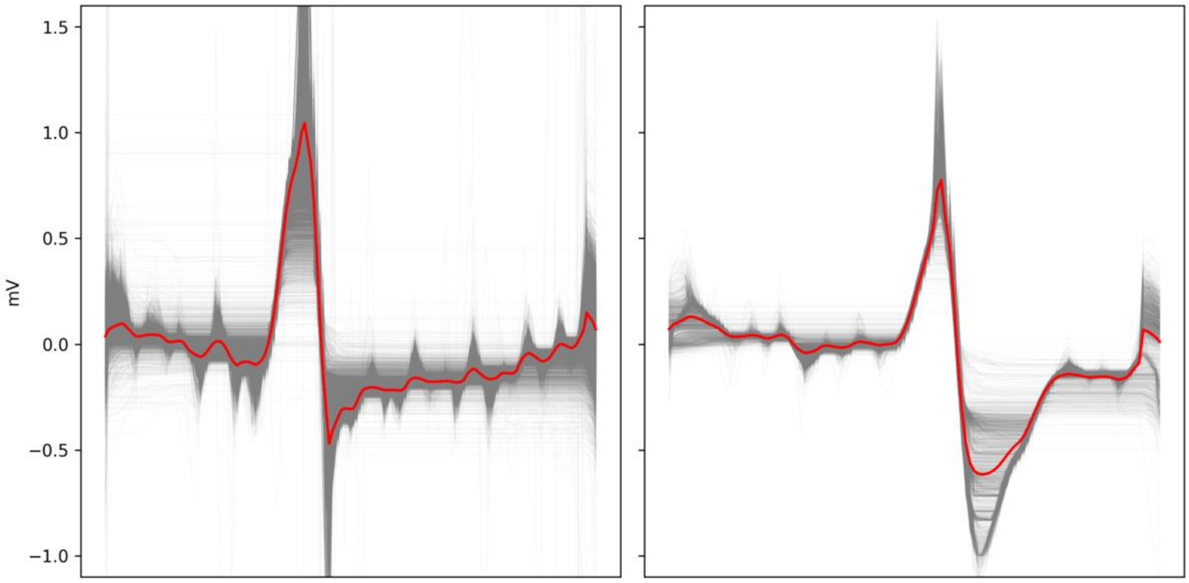

Supplementary Figure 8 | SHAP Analysis of ECG Segments. ECG waves whose QRS-complexes contribute to the prediction of absence of CAD (left) and whose ST-Segments contribute to the prediction of the presence of CAD (right). All extracted ECG waves are aligned using dynamic time warping and are shown in gray. The average wave is shown in red. There is slowed ventricular activation and distinct ST-segment depression in the mean wave associated with high SHAP values (right) as compared to patients for which a low CAD-risk was predicted (left).

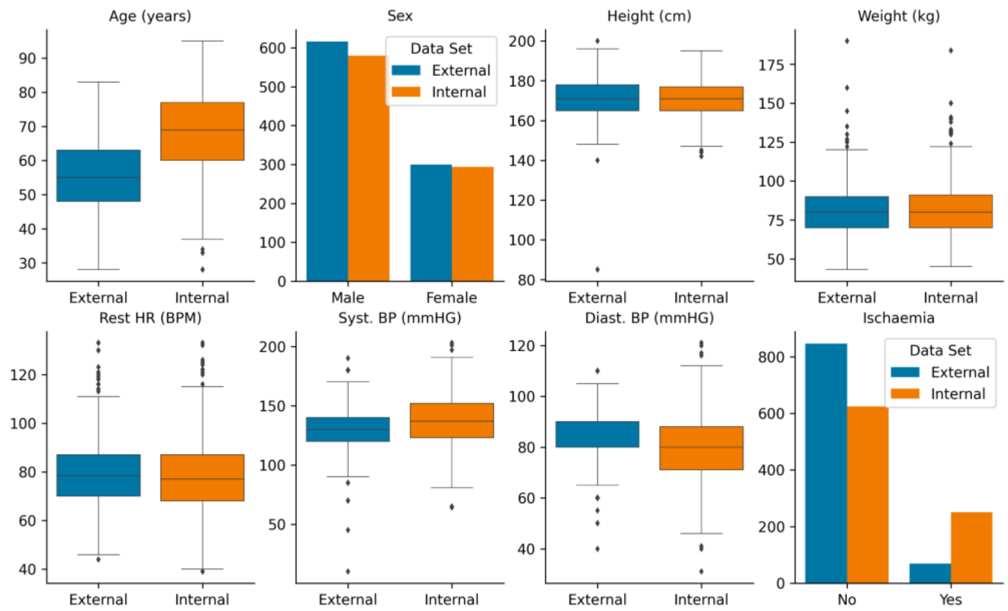

Supplementary Figure 9 | Variable Distributions. Comparing relevant clinical variables from the internal with the external data set. While most variables follow a similar distribution in both data sets, the ages of both study populations differ significantly. In addition, the prevalence of CAD in the external data set is significantly lower than in our internal data set. The bottom and top edges of the boxplots correspond to the 25th (Q1) and the 75th (Q3) percentile, respectively. The horizontal line in the center is the median value. Whiskers capture data in the 1.5x interquartile ranges beyond and below Q1 and Q3. Outliers are visualized as diamonds.

Supplementary Table 9 | Demographic and clinical characteristics of patients in development and held-out test set. Source data are provided as a Source Data file.

**Characteristic**	Development set (n=2648)	Held-out test set (n=874)
Median age (SD), years	66.7 (11.1)	68.2 (11.4)
Female, % (n)	849 (32)	294 (34)
Median body mass index [IQR], kg/m2	27.3 [24.5, 30.8]	27.2 [24.2, 30.4]
Ever smoking, % (n)	1588 (60)	554 (63)
Hypertension, % (n)	2166 (82)	694 (79)
Dyslipidemia, % (n)	1902 (72)	633 (72)
Diabetes, % (n)	684 (26)	205 (23)
Heart failure, % (n)	75 (3)	19 (2)
Atrial Fibrillation, % (n)	388 (15)	144 (16)
Pacemaker, % (n)	149 (6)	59 (7)
**CAD history**, **% (n)**	1264 (48)	393 (45)
Prior myocardial infarction, % (n)	782 (30)	235 (27)
Coronary artery bypass grafting, % (n)	354 (13)	126 (14)
PCI, % (n)	973 (37)	300 (34)
**Medications**		
ACE inhibitor, % (n)	837 (32)	267 (31)
Angiotensin-receptor antagonists, % (n)	838 (32)	280 (32)
Aspirin, % (n)	1643 (62)	515 (59)
β-Blocker, % (n)	1507 (57)	450 (51)
Calcium antagonist, % (n)	611 (23)	187 (21)
Statins, % (n)	1558 (59)	520 (59)
Nitroglycerine, % (n)	280 (11)	62 (7)
**Stress testing**		
Pure exercise stress testing, % (n)	1417 (54)	517 (59)
Pre stress testing VAS [IQR], %	40.0 [30.0, 60.0]	35.0 [20.0, 50.0]
Post stress testing VAS [IQR], %	40.0 [20.0, 70.0]	30.0 [20.0, 60.0]
Resting heart rate (SD), bpm	75.6 (15.0)	78.3 (14.4)
Resting systolic blood pressure (SD), mmHg	133.8 (22.6)	137.4 (20.5)
Resting diastolic blood pressure (SD), mmHg	80.4 (13.6)	79.7 (12.6)
**Functionally relevant CAD, % (n)**	909 (34)	250 (29)

ACE = angiotensin-converting enzyme; CAD = coronary artery disease; IQR - interquartile range; PCI = percutaneous coronary intervention.

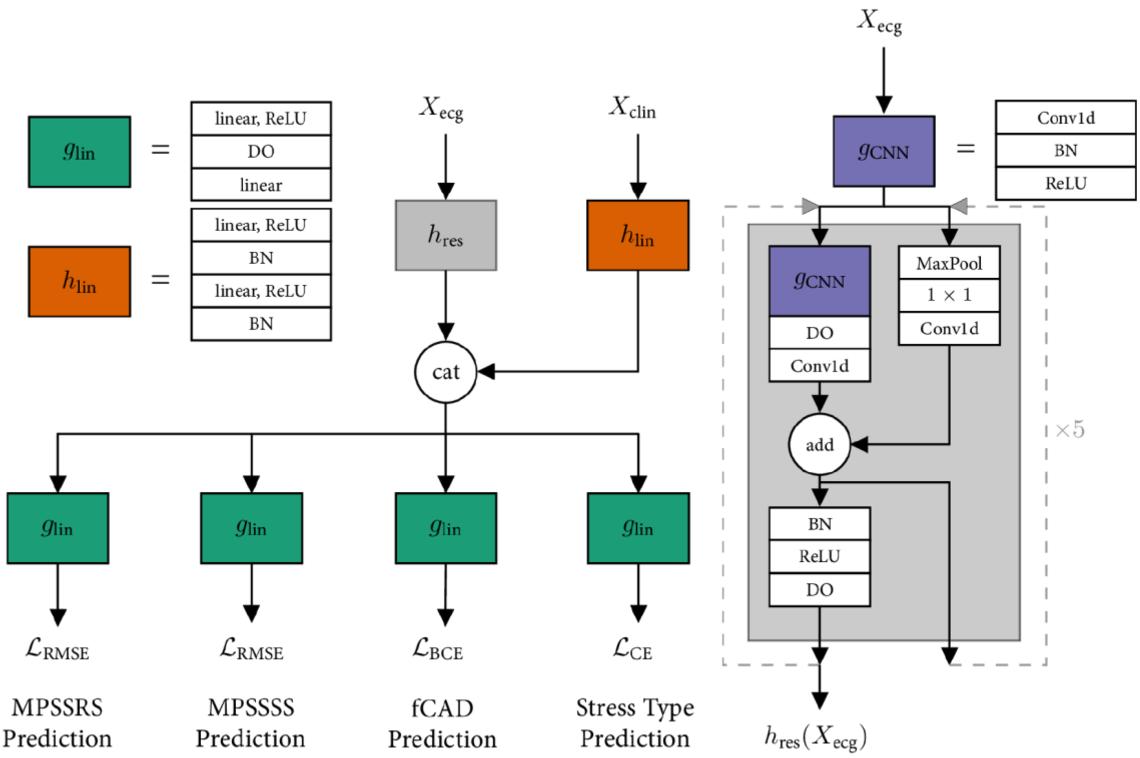

Supplementary Figure 10 | **Neural Network Architecture.** Left: Composition of our multi-task architecture. Each task obtains its own loss function *L*. RMSE: Root Mean Squared Error, BCE: Binary Cross Entropy, CE: Cross Entropy. Linear blocks are composed of linear (feedforward) layers with Rectified Linear Units (ReLU) as activation function, dropout (DO), and batch normalisation (BN). Right: Residual neural network $\mathbf{h}_{\text{res}}$ with ECG signal $\mathbf{X}_{\text{ecg}}$ as input. cat denotes the concatenation of the embeddings of the ECG signal $\mathbf{X}_{\text{ecg}}$ and the clinical data $\mathbf{X}_{\text{clin}}$. DO: Dropout, BN: Batch normalisation. Conv1d: 1-dimensional convolutional neural network layer.

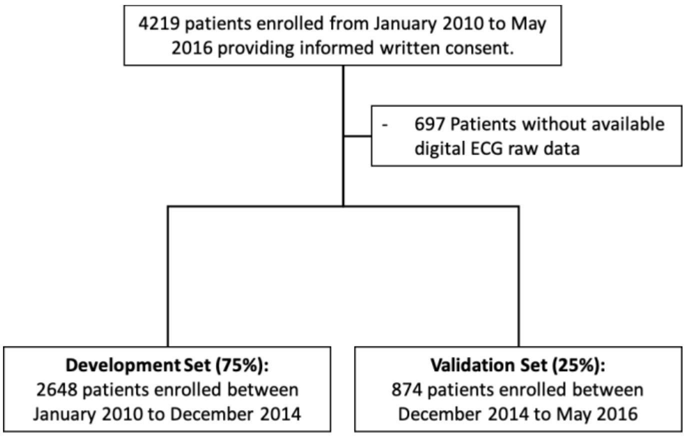

Supplementary Figure 11 | **Data Split.** After excluding all patients without available digital ECG raw data, a temporal split was performed.

Supplementary Table 10 | Parameter grid for ST-segment depression baseline. Source data are provided as a Source Data file.

Parameter	Values
Difference computation	{ST_Stress - ST_Pre, ST_Rec - ST_Pre}
Difference aggregation	{mean, median, min, max}
l_PR	{20ms, 40ms, 100ms}
