# Supplementary material for: Enhancing the diagnosis of functionally relevant coronary artery disease with machine learning
Source: Nat Commun. 2024 Jun 12;15:5034. doi: 10.1038/s41467-024-49390-y (PMC11169272; doi:10.1038/s41467-024-49390-y)
Supplement: Supplementary file 3 — Reporting Summary [file 41467_2024_49390_MOESM3_ESM.pdf]

Corresponding author(s): Karsten Borgwardt
Christian Mueller

Last updated by author(s): May 2, 2024

Reporting Summary

Nature Portfolio wishes to improve the reproducibility of the work that we publish. This form provides structure for consistency and transparency in reporting. For further information on Nature Portfolio policies, see our Editorial Policies and the Editorial Policy Checklist.

Statistics

For all statistical analyses, confirm that the following items are present in the figure legend, table legend, main text, or Methods section.

n/a	Confirmed	
☐	☒	The exact sample size (*n*) for each experimental group/condition, given as a discrete number and unit of measurement
☐	☒	A statement on whether measurements were taken from distinct samples or whether the same sample was measured repeatedly
☐	☒	The statistical test(s) used AND whether they are one- or two-sided<br>*Only common tests should be described solely by name; describe more complex techniques in the Methods section.*
☐	☒	A description of all covariates tested
☐	☒	A description of any assumptions or corrections, such as tests of normality and adjustment for multiple comparisons
☐	☒	A full description of the statistical parameters including central tendency (e.g. means) or other basic estimates (e.g. regression coefficient) AND variation (e.g. standard deviation) or associated estimates of uncertainty (e.g. confidence intervals)
☐	☒	For null hypothesis testing, the test statistic (e.g. *F*, *t*, *r*) with confidence intervals, effect sizes, degrees of freedom and *P* value noted<br>*Give P values as exact values whenever suitable.*
☒	☐	For Bayesian analysis, information on the choice of priors and Markov chain Monte Carlo settings
☒	☐	For hierarchical and complex designs, identification of the appropriate level for tests and full reporting of outcomes
☒	☐	Estimates of effect sizes (e.g. Cohen's *d*, Pearson's *r*), indicating how they were calculated

Our web collection on statistics for biologists contains articles on many of the points above.

Software and code

Policy information about availability of computer code

Data collection	The 12-lead ECG signals were recorded with two ECG machines, namely a Schiller AT-110 and Schiller CS-200 Excellence. Data was collected as .xml and .ful file through the machines' data export functionality.
Data analysis	Exported .ful and .xml files were analyzed with the standard xml module of python3.8 and numpy at version 1.24.4. ECG preprocessing was performed using the 'signal' module of scipy at version 1.10.l. QRS Delineation was performed using the matlab ecg-kit version 1.4.0.0. To train our models and analyze and visualize the data, we used the following python libraries and versions ipython==S.12.3 jupyter==l.0.0 matplotlib==3.7.3 networkx==2.8.8 notebook==7.0.4 numpy==l.24.4 pandas==2.0.3 pytorch-lightning==l.2.1 scikit-learn==l.2.2 scipy==l.10.1 seaborn==0.13.0 torch==l.6.0 torchmtl==0.1.9. Preprocessing scripts, trained neural network model checkpoints and random forest classifier are publicly available at https://github.com/BorgwardtLab/CARPE.

For manuscripts utilizing custom algorithms or software that are central to the research but not yet described in published literature, software must be made available to editors and reviewers. We strongly encourage code deposition in a community repository (e.g. GitHub). See the Nature Portfolio guidelines for submitting code & software for further information.

Data

Policy information about availability of data

All manuscripts must include a data availability statement. This statement should provide the following information, where applicable:

- Accession codes, unique identifiers, or web links for publicly available datasets
- A description of any restrictions on data availability
- For clinical datasets or third party data, please ensure that the statement adheres to our policy

The data that support some of the findings of this study are not openly available due to reasons of sensitivity of patient data and are available from the corresponding author (christian.mueller@usb.ch) upon request. The request should include the name and full contact information of the person and institution requesting the data, the specific identification of the data being requested and the purpose of requesting the data. Data requests under agreement will be considered for purposes of reproducing the data presented herein, subject to appropriate confidentiality obligations and restrictions. The timeframe for response to requests is estimated to be four to eight weeks and restrictions imposed on data use will be individualized by case-by-case data use agreements. The data resides in the secured IT infrastructure of the University Hospital Basel and respective files can be shared after anonymization upon individual request. Data used for external validation was provided by the Telemetric and Holter ECG Warehouse of the University of Rochester (THEW), NY. It cannot be made public by the authors. To obtain access, interested parties must register with the THEW project (http://thew-project.org/), submit a research proposal, and fill out the data usage agreement for the dataset with identifier E-OTH-12-0927-015. For-profit organisations may also purchase the data set for an access fee as detailed on the website. The authors declare that all data supporting the findings of this study which are not protected by patient privancy considerations, are available within the paper, its supplementary information files and downloadable files deposited at figshare (https://doi.org/10.6084/m9.figshare.25514644).

Research involving human participants, their data, or biological material

Policy information about studies with human participants or human data. See also policy information about sex, gender (identity/presentation), and sexual orientation and race, ethnicity and racism.

Reporting on sex and gender	The "sex" of the patient was based on the medical files of the University Hospital Basel which represent the Swiss civil status register (i.e., "Geschlecht/Sex" as listed on the passport). Disaggregated sex and gender data has not been collected. It can be assumed that for the vast majority of patients this represents the sex assigned at birth. Sex-based analyses were predefined and performed in this analysis.
Reporting on race, ethnicity, or other socially relevant groupings	No socially constructed or socially relevant categorization variables concerning race, ethnicity or socially relevant groupings were used in this manuscript.
Population characteristics	Please refer to "Supplementary Table 9" for details on demographic and clinical characteristics of patients in development and held-out test set in the manuscript.
Recruitment	Consecutive adult patients referred to the University Hospital Basel, Switzerland for rest/stress myocardial perfusion single-photon emission tomography/computer tomography (MPI-SPECT/CT) with symptoms possibly due to inducible myocardial ischaemia were enrolled and data collected between January 2010 to May 2016. Enrollment was embedded in routine clinical practice and proposed to every adult patient.
Ethics oversight	The Basel VIII study was approved by the local ethics committee (swissethics, BASEC, Ethikkommission Nordwest- und Zentralschweiz) under the number EKBB 100/04 and carried out according to the principles of the Declaration of Helsinki.

Note that full information on the approval of the study protocol must also be provided in the manuscript.

Field-specific reporting

Please select the one below that is the best fit for your research. If you are not sure, read the appropriate sections before making your selection.

☒ Life sciences ☐ Behavioural & social sciences ☐ Ecological, evolutionary & environmental sciences

For a reference copy of the document with all sections, see nature.com/documents/nr-reporting-summary-flat.pdf

Life sciences study design

All studies must disclose on these points even when the disclosure is negative.

Sample size	The sample size for our study was determined by the availability of high-quality data from the clinical trial with clinicaltrials.gov ID NCT01838148. At study begin, we considered the data of 4219 patients who enrolled into the clinical trial between Jan. 2010 and May 2016. After removing 697 patients due to data quality issues, we performed a temporal data split in a 3:1 which results in a sufficiently high number of training examples and a large enough validation cohort to measure statistical effects. More specifically, we trained our machine learning models on the data from 2648 patients who enrolled between Jan. 2010 and Dec. 2014. Upon completion of model training, we evaluated the predictive performance of our system on 803 (after removal of patients with missing variables) patients who enrolled between Dec. 2014 and May 2016. While 2648 patients can be considered a small sample size in the context of the training of deep learning models, we apply an ECG up-sampling scheme which increases the number of training samples by approximately 20 times.

Data exclusions	697 patients were excluded because no digital ECG raw data was available. Furthermore, 71 patients were excluded from our final performance evaluation due to missing variables. In the external data set, 11 out of 927 patients were excluded due to missing variables.
Replication	On each of the 5 data data splits, we trained one model to get uncertainty estimates. After training, all parameters are fixed and subsequent analyses are deterministic and can be replicated successfully.
Randomization	No randomization took place as our study is no RCT but a retrospective study which does not require patients to be split into different experimental groups. However, for model development, we split the data set 3:1 into a development and held-out test set containing 2648 and 874 patients, respectively. The split was performed on a temporal basis with 2648 patients enrolled between January 2010 and December 2014 (development set) and 874 patients enrolled between December 2014 and May 2016 (held-out test set). The development set was further divided into 5 stratified (i.e., fCAD prevalence in each split are the same) splits of training, validation, and calibration set, where the latter makes up 10 % of the training set. The ratio of training to validation set size is 4:1.
Blinding	In the process of model development, access to the held-out test set was restricted to prevent the models from being tailored to an unseen cohort. During the data collection phase, investigators were blinded to the group allocations (model development and internal validation). However, blinding during the analysis phase was not practicable due to the inherent nature of the tasks involved in model development versus model validation. More precisely, once model development concluded (based on performance on the development set), no additional blinding is required to investigate the model's generalization capabilities on the held-out test set. In addition, the investigators were blinded to the external validation data set during model development. Lastly, representing clinical practice, adjudication of functionally relevant CAD was not formally blinded for stress ECG results or demographics and was performed centrally by an expert team composed of a nuclear medicine physician and a cardiologist assessing myocardial perfusion scans.

Reporting for specific materials, systems and methods

We require information from authors about some types of materials, experimental systems and methods used in many studies. Here, indicate whether each material, system or method listed is relevant to your study. If you are not sure if a list item applies to your research, read the appropriate section before selecting a response.

Materials & experimental systems

n/a	Involved in the study	
☒	☐	Antibodies
☒	☐	Eukaryotic cell lines
☒	☐	Palaeontology and archaeology
☒	☐	Animals and other organisms
☐	☒	Clinical data
☒	☐	Dual use research of concern
☒	☐	Plants

Methods

n/a	Involved in the study	
☒	☐	ChIP-seq
☒	☐	Flow cytometry
☒	☐	MRI-based neuroimaging

Clinical data

Policy information about clinical studies

All manuscripts should comply with the ICMJE guidelines for publication of clinical research and a completed CONSORT checklist must be included with all submissions.

Clinical trial registration	NCT01838148, clinicaltrials.gov
Study protocol	The complete study protocol of the Biochemical and Electrocardiographic Signatures in the Detection of Exercise-induced Myocardial Ischemia (BASEL VIII) can be made available upon request. At our institution it is not commonplace to publish the complete study protocol of a prospective diagnostic study.
Data collection	Consecutive adult patients referred to the University Hospital Basel, Switzerland for rest/stress myocardial perfusion single-photon emission tomography/computer tomography (MPI-SPECT/CT) with symptoms possibly due to inducible myocardial ischaemia were enrolled and data collected between January 2010 to May 2016.
Outcomes	Adjudication of fCAD was based on expert interpretation of MPI-SPECT/CT images combined with information obtained from invasive coronary angiography and fractional flow reserve measurements, whenever available. All patients underwent a routine rest/stress dual isotope (201Tl for rest, 99mTc sestamibi for stress) or a single isotope (99mTc sestamibi for stress and rest) MPI-SPECT/CT protocol as described previously24-26. MPI-SPECT/CT images were scored semi-quantitatively using a 17-segment model with a 5-point scale (0=normal, 1=mildly reduced tracer uptake, 2=moderately reduced uptake, 3=severely reduced uptake and 4=no uptake). Summed stress score and summed rest score were calculated by adding the scores of the 17 segments in the stress and rest images. Summed difference score was the difference between summed stress score and summed rest score. A summed difference score of at least 2 or positive transient ischemic dilation ratio (≥1.22 for the dual isotope protocol and 1.12 for the single isotope protocol) was considered as fCAD. Summed stress score and summed rest score were derived by visual assessment of two expert readers and compared with the software result. Differences in the visual assessment were resolved by finding consensus. In case of equivocal findings from MPI-SPECT/CT and coronary angiography, an adjudication committee of two independent cardiologists (one interventional cardiologist, one general cardiologist) that were blinded to biomarker results reviewed the case. A positive perfusion scan was overruled when coronary angiography showed normal coronary arteries, while a negative perfusion scan was overruled if coronary angiography (within three months) either revealed a high-grade coronary lesion (>75%) or if there was fractional flow

reserve below 0.80.

Plants

Seed stocks

Report on the source of all seed stocks or other plant material used. If applicable, state the seed stock centre and catalogue number. If plant specimens were collected from the field, describe the collection location, date and sampling procedures.

Novel plant genotypes

Describe the methods by which all novel plant genotypes were produced. This includes those generated by transgenic approaches, gene editing, chemical/radiation-based mutagenesis and hybridization. For transgenic lines, describe the transformation method, the number of independent lines analyzed and the generation upon which experiments were performed. For gene-edited lines, describe the editor used, the endogenous sequence targeted for editing, the targeting guide RNA sequence (if applicable) and how the editor was applied.

Authentication

Describe any authentication procedures for each seed stock used or novel genotype generated. Describe any experiments used to assess the effect of a mutation and, where applicable, how potential secondary effects (e.g. second site T-DNA insertions, mosiacism, off-target gene editing) were examined.
